# Identification of a Divergent Avian Influenza H3N2 Virus from Domestic Ducks in France

**DOI:** 10.1128/MRA.00943-18

**Published:** 2018-12-13

**Authors:** François-Xavier Briand, Eric Niqueux, Audrey Schmitz, Veronique Beven, Pierrick Lucas, Chantal Allée, Marina Chatel, Fabrice Touzain, Yannick Blanchard, Nicolas Eterradossi

**Affiliations:** a Anses, Unité VIPAC–LNR Influenza Aviaire, Ploufragan, France; b Anses, Unité Génétique Virale et Biosécurité, Ploufragan, France; c Université Bretagne-Loire, Rennes, France; Indiana University, Bloomington

## Abstract

An avian influenza H3N2 virus was isolated from domestic ducks in France in 2016. Although this French H3N2 virus possesses traits of an avian virus, the genetic distances observed for hemagglutinin (HA) and neuraminidase (NA) show that these two genes most likely evolved independently from other avian influenza sequences.

## ANNOUNCEMENT

Avian influenza A virus (AIV) is a single-stranded multisegmented negative-sense RNA virus belonging to the Alphainfluenzavirus genus, family *Orthomyxoviridae* (1). Wild aquatic birds are a reservoir of AIVs and play a major epidemiological role in their persistence and spread along migratory flyways (2). AIVs are classified into subtypes based on antigenic differences in their hemagglutinin (HA) and neuraminidase (NA) proteins. AIVs mostly cause mild respiratory, digestive, and genital infections in birds; however, mutations in the H5 and H7 subtypes can confer high pathogenicity (HP). Despite some host range restriction, AIVs infect a variety of wild and domestic birds and can cross the species barrier to infect mammals (e.g., humans, pigs, and horses) (3). For example, H3N2 viruses have crossed the species barrier to settle in human and swine hosts (4).

During the winter of 2015 to 2016, southwestern France was confronted with a large H5 HP epizootic outbreak, with 81 cases mainly detected in duck farms (5). To limit its spread, molecular monitoring was implemented in domestic ducks. Viral RNA extracted from cloacal and tracheal swabs was tested using M gene and H5 gene real-time reverse transcription-PCRs (rRT-PCRs). The overall diversity of the detected AIVs is under investigation; however, the characterization of the H5-positive samples revealed high genetic diversity (5). Among cloacal samples which tested M positive and H5 negative, one H3N2 virus was detected. This virus, A/duck/France/161005/2016, was isolated in embryonated specific-pathogen-free (SPF) chicken eggs. Viral RNA was extracted from allantoic fluid, and AIV segments were amplified by RT-PCR using specific primers matching 12 or 13 nucleotides at the 5′ and 3′ conserved ends of the AIV genome segments (6). The resulting amplicons, covering the entire genome except the outermost nucleotides of each genome segment, were sequenced by next-generation sequencing (NGS) using the Ion Torrent Proton system. More than 424,300 reads were obtained before cleaning. Their average length was 109 nucleotides. Cleaning, quality control, and assembly were performed using Trimmomatic 0.32 (7), FastQC (8), and SPAdes 3.10.0 (9), respectively.

**FIG 1:**
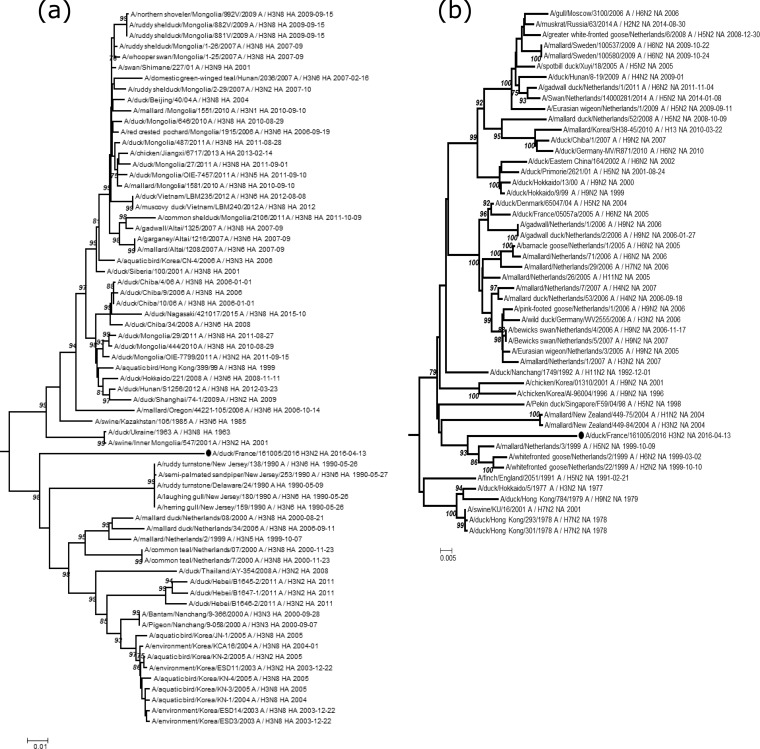
Partial phylogenetic tree of the H3 (a) and N2 (b) genes and their closest related sequences. The outgroups were HA and NA sequences from human H3N2. Trees were generated using the neighbor-joining method and Kimura 2-parameter model with 1,000 bootstrap replicates. Only bootstrap values superior to 75 are indicated. The analyses were based on 1,550 and 1,312 nucleotides for HA and NA, respectively. French H3N2 sequences are indicated by a black dot.

Internal gene segments (PB2/PB1/PA/NP/M/NS) were close to sequences from European avian viruses, with nucleotide identities ranging from 97.1% to 99.1%. Discrepantly, the HA and NA segments exhibited more nucleotide differences, with their closest genetic relatives having 89.1% and 94.4% identity, respectively. Phylogenetic analyses by neighbor joining (MEGA 7.0 software [10]) showed that the HA sequence was related to a small cluster comprising avian European and Asian H3 sequences detected from 1999 to 2011 and avian North American H3 sequences detected in 1990 (Fig. 1a). Although the newly detected HA sequence was quite different from other avian H3 sequences, it still exhibited amino acids Q226 and G228, which have been shown to be essential for HA binding to the specific avian alpha 2-3 receptor (11). In comparison, phylogenetic analyses of the French N2 gene indicated that it was related to a cluster composed only of avian Dutch sequences (Fig. 1b). Although this French H3N2 virus possesses traits of an avian virus, the genetic distance observed for HA, and to a lesser extent for NA, shows that these two genes most likely evolved independently from other avian influenza sequences and possibly were incorporated into the genome by one or more reassortments. Increased sampling for AIV surveillance from domestic or wild ducks is essential to better understand the genetic evolution of AIVs and to possibly detect the emergence of mutations involved in crossing the species barrier.

### Data availability.

Reads can be accessed through the Sequence Read Archive (SRA) database (under BioProject number PRJNA490079). The A/duck/France/161005/2016 (H3N2) viral coding sequence was submitted to the GenBank database under accession numbers MH560268 through MH560275.
